# Clinical Utility of 18F-FDG PET/CT in Rheumatology: Diagnostic and Therapeutic Insights from a Ten-Year Real-World Cohort †

**DOI:** 10.3390/jcm15051872

**Published:** 2026-02-28

**Authors:** Mert Can Ataca, Semih Gulle, Yesim Erez, Erkan Derebek, Gercek Sen

**Affiliations:** 1 Department of Internal Medicine, Faculty of Medicine, Dokuz Eylul University, Izmir 35340, Turkey; 2 Department of Internal Medicine, Division of Rheumatology, Faculty of Medicine, Dokuz Eylul University, Izmir 35340, Turkey; 3 Department of Nuclear Medicine, Faculty of Medicine, Dokuz Eylul University, Izmir 35340, Turkey

**Keywords:** PET-CT, rheumatology, vasculitis, IgG4-related disease, sarcoidosis, diagnostic imaging

## Abstract

**Objective:** To evaluate the diagnostic yield and clinical impact of 18F-fluorodeoxyglucose positron emission tomography/computed tomography (18F-FDG PET/CT) in patients with unexplained inflammation, fever, or suspected malignancy, and to assess its role across different rheumatologic subgroups. **Methods:** We retrospectively analyzed 280 patients who underwent PET/CT between 2010 and 2020 in a tertiary rheumatology center. Demographic, clinical, and laboratory data including erythrocyte sedimentation rate, C-reactive protein, and PET/CT indications were collected. PET/CT findings were categorized as inflammatory, neoplastic, or normal based on visual assessment and SUVmax. Final diagnoses were confirmed using clinical, histopathological, or follow-up data. Statistical analysis compared PET/CT results, inflammatory markers, and diagnostic outcomes among disease subgroups. **Results:** Of 280 patients (mean age 58 ± 15 years, 63.9% female), 72% had an established rheumatologic diagnosis prior to PET/CT. A new diagnosis, confirmed by predefined clinical, histopathological, or follow-up criteria, was established in 29.6% of patients, predominantly among those undergoing diagnostic evaluation for unexplained inflammation, including 40 rheumatologic and 43 non-rheumatologic conditions (22 malignancies). PET/CT led to therapeutic modification in 27.1% of all cases, based on multidisciplinary clinical decision-making. PET/CT demonstrated the highest diagnostic contribution in vasculitis, IgG4-related disease, and sarcoidosis. Median SUVmax was higher in malignancies than in inflammatory diseases [8.0 vs. 4.6, *p* < 0.05]. Lymphadenopathy was more frequent in non-rheumatologic and malignant conditions (*p* = 0.002). PET/CT findings showed variable but clinically relevant concordance with other imaging modalities. **Conclusions:** PET/CT provides supportive diagnostic and management insights in complex or atypical rheumatologic presentations. It demonstrated high yield in systemic inflammatory disorders and providing supportive information for malignancy exclusion in connective tissue diseases when interpreted alongside clinical and laboratory follow-up. Integration of PET/CT with clinical and laboratory data enhances diagnostic accuracy and supports patient-centered management in rheumatology.

## 1. Introduction

Rheumatologic diseases encompass a broad spectrum of autoimmune and autoinflammatory disorders characterized by chronic systemic inflammation and progressive tissue damage. While clinical assessment remains central to diagnosis, laboratory and imaging modalities provide critical adjunctive information for disease characterization and monitoring [1]. Conventional imaging techniques such as radiography, ultrasonography, computed tomography (CT), and magnetic resonance imaging (MRI) primarily assess structural damage or localized inflammation, but they offer limited insight into systemic inflammatory activity. PET/CT enables visualization of metabolically active inflammatory lesions throughout the body, offering a sensitive and noninvasive approach to detect and quantify inflammation. Originally developed for oncologic imaging, PET/CT has increasingly demonstrated value in the evaluation of systemic inflammatory and autoimmune diseases [2,3]. Beyond its diagnostic role, it can objectively assess treatment response through changes in FDG uptake, providing a dynamic measure of disease activity. PET/CT is particularly informative in patients with fever or inflammation of unknown origin, in whom conventional investigations fail to localize the underlying process.

The diagnostic utility of PET/CT is well established in large-vessel vasculitides such as giant cell arteritis (GCA) and Takayasu arteritis. Recent studies have expanded its use to other systemic rheumatic diseases, including polymyalgia rheumatica (PMR), sarcoidosis, IgG4-related disease, rheumatoid arthritis (RA), and connective tissue diseases (CTDs) [4,5,6]. Reported diagnostic yields in fever or inflammation of unknown origin range from 38% to 75% [7,8], but most data derive from internal medicine or infectious disease cohorts rather than dedicated rheumatology populations. Moreover, despite its high sensitivity, the relatively low specificity of PET/CT necessitates careful integration with clinical, serologic, and histopathologic data to differentiate inflammatory from malignant etiologies.

To address these gaps, we conducted a comprehensive real-world analysis of patients undergoing 18F-FDG PET/CT in a tertiary rheumatology center over a ten-year period. The study aimed to evaluate the diagnostic yield, sensitivity, and clinical impact of PET/CT in patients with unexplained systemic inflammation or suspected malignancy, and to assess its role in disease activity monitoring and treatment decision-making across diverse rheumatologic subgroups. By characterizing the patterns of PET/CT utilization and its contribution to diagnosis and therapy, this study provides novel insights into the expanding role of PET/CT in rheumatologic practice.

## 2. Materials and Methods

### 2.1. Study Design and Population

This retrospective study included patients aged ≥18 years who underwent 18F-FDG PET/CT in the outpatient or inpatient rheumatology units of Dokuz Eylul University between 1 January 2010, and 31 December 2020. Eligible patients had complete clinical and laboratory data available at the time of imaging. Demographic characteristics, comorbidities, clinical presentations (e.g., including fever at admission), erythrocyte sedimentation rate (ESR), and C-reactive protein (CRP) levels were extracted from medical records.

Exclusion criteria were: age < 18 or >80 years, history of malignancy, active infection, pregnancy or lactation, and recent major trauma, surgery, or hemorrhage. Active infection was defined as any ongoing infectious process confirmed by clinical evaluation, laboratory markers, or other imaging modalities. Patients with no evidence of overt infection based on these assessments were eligible for inclusion. As this study adhered to STROBE reporting guidelines for observational research, a formal flow diagram was not generated. The patient selection process, including all inclusion and exclusion criteria, is described in detail above. This approach reflects an unselected, real-world rheumatology PET/CT population.

### 2.2. Data Collection and Classification

Clinical data included demographic features, underlying rheumatologic diagnoses, medications, and PET/CT indications (e.g., suspected vasculitis, malignancy, or fever of unknown origin). PET/CT reports were reviewed by an experienced nuclear medicine specialist and classified as normal, inflammatory, or suggestive of malignancy, based on both visual interpretation and maximum standardized uptake values (SUVmax).

Complementary radiologic, serologic, and histopathologic evaluations were reviewed to determine final diagnoses, which were categorized as autoimmune, infectious, neoplastic, or undiagnosed. For unresolved cases, follow-up data, including empirical treatments and serial inflammatory markers, were assessed.

Laboratory and treatment data within four weeks prior to PET/CT were recorded, prioritizing values closest to the imaging date. Immunologic markers were also documented in temporal proximity to PET/CT acquisition. Corresponding findings from other imaging modalities (MRI, CT angiography, Doppler ultrasonography) and follow-up PET/CT scans were included for concordance analysis.

### 2.3. PET/CT Protocol

All patients fasted for at least six hours before imaging, with serum glucose confirmed at <200 mg/dL. After intravenous administration of 7–15 mCi (259–555 MBq) of 18F-FDG (Monrol, Eczacıbaşı, Istanbul, Turkey), imaging was performed approximately one hour later.

Low-dose CT scans were first obtained for attenuation correction, followed by PET acquisitions from the proximal thighs to the skull vertex in the supine position. Imaging was performed using a Philips Gemini TF PET/CT scanner (Philips Healthcare, Cleveland, OH, USA) in 3D mode (spatial resolution 4.7 mm; LYSO crystal, 4 × 4 × 22 mm; 28,336 crystals). CT parameters were 50–100 mAs, 120 kVp, 5 mm slice thickness, 0.5 s rotation time, and 39 mm/s table speed. PET data were acquired over 6–7 bed positions, with an acquisition time of 1.5–2 min per bed.

Image reconstruction was performed using an iterative algorithm. The resulting images were evaluated both visually and semi-quantitatively based on SUVmax.

Standardized uptake values were calculated as follows:

SUV = Activity concentration in ROI (mCi/mL)/[Injected dose (mCi)/Body weight (kg)]

SUVmax, defined as the highest voxel value within the region of interest, was used for analysis due to its reproducibility and independence from observer variability.

### 2.4. Image Interpretation and Bias Mitigation

While images were initially interpreted by a single experienced nuclear medicine specialist in a real-world clinical setting, a retrospective review of a representative subset was performed by a second blinded reader to assess inter-observer agreement. For large-vessel vasculitis, although SUVmax was the primary metric, visual grading was cross-referenced with the liver and mediastinal blood pool to ensure standardized activity assessment.

### 2.5. Operational Definitions and Diagnostic Criteria

The diagnostic contribution of 18F-FDG PET/CT was defined as the identification of a new clinical entity that was not previously detected by conventional imaging or physical examination. A PET/CT finding was considered ‘diagnostic’ if it led to a final diagnosis confirmed by: (a) histopathological evidence from a PET-guided biopsy, (b) characteristic imaging patterns combined with a clinical and laboratory response to specific therapy, or (c) consistent findings during at least 6 months of clinical follow-up. Disease subgroups were classified according to international criteria: the 2022 ACR/EULAR criteria for Giant Cell Arteritis (GCA) and Takayasu Arteritis (TAK), the 2019 ACR/EULAR classification criteria for IgG4-related disease, and the 1991 Revised ACE criteria for Sarcoidosis.

### 2.6. Statistical Analysis

All analyses were conducted using SPSS version 26.0 (IBM Corp., Armonk, NY, USA). Data normality was assessed with the Shapiro–Wilk Francia test, and variance homogeneity with Levene’s test. Between-group comparisons of continuous variables were performed using the independent-samples *t*-test (with bootstrap results) or the Mann–Whitney *U* test (with Monte Carlo simulation) as appropriate.

Categorical variables were compared using the Pearson χ^2^ test, Linear-by-Linear Association, and Fisher–Freeman–Halton tests with Monte Carlo simulation, or Fisher’s exact test when applicable. Column ratios were compared using Benjamini–Hochberg correction for multiple testing. To account for the heterogeneity of the study population, separate sub-analyses were conducted for patients with established diagnoses versus those undergoing initial diagnostic work-up. Multivariate logistic regression was performed to identify independent predictors of ‘new diagnosis’ and ‘treatment modification,’ adjusting for age, gender, baseline CRP levels, and current glucocorticoid use. Continuous variables are presented as mean ± standard deviation (SD) or median [interquartile range (IQR)], and categorical variables as *n* (%). A *p*-value < 0.05 was considered statistically significant. Bonferroni correction was applied to all multiple comparisons, including subgroup analyses and SUVmax comparisons, to control for type I error; corrected *p*-values are reported accordingly.

### 2.7. Ethical Approval

This study was conducted in accordance with the Declaration of Helsinki and approved by the Dokuz Eylul University Non-Interventional Clinical Research Ethics Committee (Non-Interventional Clinical Research Decision No: Approval 2021/07-24/1 March 2021, File No: 6139-GOA). Due to the retrospective design, the requirement for informed consent was waived.

## 3. Results

### 3.1. Demographic Characteristics

A total of 280 patients were included (mean age 58 ± 15 years; 63.9% female). At the time of PET/CT, 202 patients (72.1%) had an established rheumatologic diagnosis, while 78 (27.9%) were undiagnosed. Following evaluation, 247 patients (88.2%) were classified as having a rheumatologic disease and 33 (11.8%) remained without a definitive rheumatologic diagnosis. The most frequent comorbidities were hypertension (20.9%), diabetes mellitus (11.2%), coronary artery disease (10.5%), and chronic kidney disease (5.1%).

### 3.2. Spectrum of Rheumatologic Diseases

Among the 247 patients with rheumatologic disease, connective tissue diseases (*n* = 70, 28.3%) and vasculitides (*n* = 63, 25.5%) were most common, followed by rheumatoid arthritis (*n* = 28, 11.3%) and spondyloarthropathies (*n* = 21, 8.4%). Less frequent diagnoses included IgG4-related disease (*n* = 14, 5.6%), sarcoidosis (*n* = 14, 5.6%), retroperitoneal fibrosis (*n* = 8, 3.2%), and polymyalgia rheumatica (*n* = 8, 3.2%). Rare conditions such as crystal arthropathies, autoinflammatory syndromes, and various overlapping or atypical presentations were also observed (Supplemental Figure S1).

### 3.3. Laboratory Findings and Diagnostic Yield

Median ESR and CRP levels prior to PET/CT were 43 mm/h (range 1–120) and 16.9 mg/L (range 0.2–431), respectively. Patients without a pre-existing rheumatologic diagnosis had significantly higher ESR and CRP than those with one (*p* = 0.006 and *p* = 0.024). No significant differences were observed in other laboratory parameters (Table 1).

PET/CT established a new diagnosis in 83 patients (29.6%), including 40 rheumatologic and 43 non-rheumatologic conditions (22 malignancies, 21 infections). Patients with new diagnoses had higher inflammatory markers compared to undiagnosed cases (median CRP = 33 mg/L vs. 14.3 mg/L, *p* = 0.010; ESR = 56 mm/h vs. 38 mm/h, *p* < 0.001) (Table 2). There were no significant associations between elevated CRP and diagnostic category (rheumatologic, non-rheumatologic, or malignant) or presence of lymphadenopathy (Table 3).

### 3.4. Indications for PET/CT

The main indications were malignancy work-up (*n* = 165, 58.9%), diagnostic evaluation of suspected rheumatologic disease (*n* = 91, 32.5%), and treatment-response assessment (*n* = 24, 8.6%). The most common specific triggers were lymphadenopathy (32.1%), pulmonary nodules (16.8%), and unexplained inflammation (13.2%) (Supplementary Figure S2).

Among patients with a pre-existing rheumatologic diagnosis, PET/CT was performed to exclude malignancy was requested in 65.8%, compared with 38.7% among those without a rheumatologic diagnosis (*p* < 0.001). Similarly, PET/CT due to lymphadenopathy was 34.7% vs. 26.7% in patients with vs. without a rheumatologic diagnosis (*p* < 0.001).

Malignancy was diagnosed in 22 patients, most frequently lung cancer (*n* = 8), followed by lymphoma (*n* = 3), breast cancer (*n* = 3), and pancreatic cancer (*n* = 2). The majority of malignancies (77.2%) occurred in patients with an established rheumatologic condition.

### 3.5. PET/CT Findings and SUVmax Distribution

The median SUVmax across the cohort was 3.4 (range: physiological–27.1). Patients with malignancy had significantly higher SUVmax compared to those with newly diagnosed rheumatologic disease [8.0 (6.1–10.6) vs. 4.6 (3.3–7.2), *p* < 0.05]. No significant differences in ESR, CRP, or comorbidity burden were detected between the malignancy and rheumatologic subgroups (Table 4).

Lymphadenopathy was detected in 45% of patients who received a rheumatologic diagnosis via PET/CT, compared with 74.4% of those with non-rheumatologic conditions (*p* = 0.002).

### 3.6. Disease-Specific Subgroups

Vasculitis: PET/CT contributed to diagnosis in 17.5% of cases, most commonly by demonstrating large-vessel wall uptake (Supplementary Figure S3). Glucocorticoid use at the time of imaging was reported in 68.3% and csDMARD use in 42.8% of patients. In giant cell arteritis, the main indication was diagnostic support (83.3%), whereas in Takayasu arteritis, half of the scans were performed for treatment-response assessment. Large-vessel FDG uptake was observed in 44.4% of GCA and 28% of Takayasu cases.

Polymyalgia Rheumatica: PET/CT confirmed the diagnosis in one of eight patients, while the remainder were evaluated for initial disease assessment (Supplementary Figure S4).

Connective Tissue Diseases: The predominant indication was malignancy exclusion (94.3%). FDG uptake was most frequently observed in lymph nodes (50.8%) and pulmonary nodules (20.3%). Among systemic lupus erythematosus (SLE) cases (*n* = 16), lymph node uptake occurred in 81.2%, attributed to disease activity in 76.9%. Similar findings were seen in Sjögren’s syndrome (*n* = 16), where lymph node uptake occurred in 68.7% and was attributed to activity in 81.1%.

IgG4-Related Disease and Retroperitoneal Fibrosis: PET/CT was diagnostic in 71.4% of IgG4-related disease cases, with predominant soft-tissue uptake (50%) (Supplementary Figure S5). All retroperitoneal fibrosis patients (*n* = 8) underwent PET/CT for diagnostic purposes, with soft-tissue uptake in 75%.

Sarcoidosis: PET/CT supported the diagnosis in 9 of 14 patients (64.3%), with lymph node uptake present in 92.3% and histopathologic confirmation in nine cases (Supplementary Figure S6 and Table S1).

### 3.7. Follow-Up PET/CT and Impact on Management

Follow-up PET/CT was performed in 18 patients (6.4%), predominantly for treatment monitoring (88.8%), most often in vasculitis and IgG4-related disease. Among these, therapy was modified in 4 patients. Overall, PET/CT findings (baseline and/or follow-up) led to treatment modification in 76 patients (27.1%). Treatment modification following PET/CT primarily involved initiation or escalation of immunosuppressive therapy, including glucocorticoids, conventional synthetic DMARDs, or biologic DMARDs, based on multidisciplinary clinical decision-making. Due to the retrospective design and variability in documentation, treatment changes were not further subclassified into discrete categories.

Multivariate logistic regression analysis was performed adjusting for age, gender, baseline CRP levels, and current glucocorticoid use to identify independent predictors of new diagnosis and treatment modification following PET-CT. None of the variables included in the model were independently associated with either outcome (all *p* > 0.05). These findings suggest that diagnostic refinement and treatment modification were not independently predicted by baseline demographic or inflammatory parameters in this cohort.

### 3.8. Concordance with Other Imaging Modalities

Across vasculitis subgroups, PET/CT showed variable concordance with CT angiography, MR angiography, and Doppler ultrasonography. Agreement was highest for giant cell arteritis and lowest for Takayasu arteritis (Table 5).

## 4. Discussion

In this large real-world cohort, 18F-FDG PET/CT demonstrated substantial diagnostic and clinical impact in patients with atypical or complex inflammatory presentations. The modality identified previously unrecognized rheumatologic disease in nearly one-third of cases with unexplained fever, lymphadenopathy, or elevated acute-phase reactants, while also assisting with malignancy exclusion. Importantly, PET/CT findings prompted diagnostic clarification or treatment modification in approximately 30% of patients, underscoring its relevance in both initial evaluation and disease monitoring [9].

Consistent with prior EULAR surveys, the predominant indications for PET/CT were vasculitis, fever of unknown origin (FUO), and unexplained inflammatory activity. In our study, malignancy exclusion and lymphadenopathy were the leading reasons for referral. Given the higher malignancy risk among rheumatologic patients, earlier use of PET/CT in those with unexplained inflammation or nodal involvement appears clinically justified [10,11].

Comparison with previous cohorts reveals several distinctions. Unlike the study by Schönau et al. [12], in which no patient had a prior rheumatologic diagnosis, 72% of our patients did, with many already receiving corticosteroids (53.2%) or DMARDs (38.2%). This likely contributed to lower baseline inflammatory markers and attenuated FDG uptake. Similarly to Öğüt et al. [8], PET/CT yielded new diagnoses in 74% of previously undiagnosed patients and revealed additional pathology in 12% with established disease, confirming its broad diagnostic reach.

Lymphadenopathy proved to be a key determinant of diagnostic outcome. It was more frequent in non-rheumatologic or malignant conditions, consistent with prior evidence linking nodal FDG uptake to neoplasia [8,12]. Although median SUVmax was significantly higher in malignant diagnoses compared to inflammatory conditions (8.0 vs. 4.6, *p* = 0.016), the discriminatory utility of this metric at the individual patient level is substantially limited by the considerable overlap in value distributions. Inflammatory diseases such as sarcoidosis and active large-vessel vasculitis can exhibit SUVmax values within ranges conventionally associated with malignancy, thereby reducing specificity when SUVmax is applied in isolation. These findings are consistent with prior reports demonstrating that, while SUVmax differences achieve statistical significance at the group level, no reliable individual-level threshold has been established to distinguish malignant from inflammatory FDG uptake [13,14]. Accordingly, SUVmax should be regarded as a supportive quantitative parameter rather than a standalone diagnostic discriminator, and its interpretation must be integrated with anatomical imaging characteristics, clinical context, and histopathological confirmation where indicated.

In large-vessel vasculitis, PET/CT accurately reflected vascular inflammation and disease activity, in agreement with prior reports [15,16]. Characteristic patterns in polymyalgia rheumatica and the frequent coexistence of vascular uptake emphasize its value for detecting subclinical large-vessel involvement [17,18,19]. In connective tissue diseases such as SLE and Sjögren’s syndrome, PET/CT effectively characterized lymphadenopathy and organ involvement, but differentiation from malignancy remained challenging, consistent with previous observations [20].

In contrast, the role of PET/CT in rheumatoid arthritis and spondyloarthritis was more limited but remained valuable for evaluating reactive lymphadenopathy, malignancy exclusion, and monitoring disease activity. FDG uptake in RA correlated with inflammatory markers and disease activity, aligning with previous reports, and has been shown to decrease following effective therapy. Importantly, lymphadenopathy in RA patients underscores the need for histopathological evaluation due to an increased risk of lymphoma and other malignancies [11,21,22,23].

The diagnostic yield was particularly high in IgG4-related disease, where PET/CT delineated multi-organ involvement and guided biopsy, even in cases with low inflammatory markers [24,25]. These results support the use of PET/CT for mapping organ involvement and optimizing tissue sampling in this diagnostically demanding entity.

Collectively, these findings highlight PET/CT as a powerful adjunct in the diagnostic and management algorithm of systemic inflammatory diseases. When interpreted alongside clinical, laboratory, and histopathological data, PET/CT enhances diagnostic accuracy, identifies occult inflammation, and informs therapeutic decision-making, particularly in diagnostically challenging scenarios.

This study was primarily designed as a descriptive, real-world cohort analysis aiming to characterize patterns of PET/CT utilization, diagnostic yield, and clinical impact in a heterogeneous tertiary rheumatology population. Given the broad spectrum of underlying diagnoses, variable disease activity states, and clinician-driven decision-making processes, the principal objective was not to construct a predictive model but rather to provide an observational overview of PET/CT performance across routine clinical scenarios. Therefore, descriptive statistics form the methodological backbone of the present study, while multivariate modeling was performed in an exploratory manner to assess whether baseline demographic or inflammatory parameters independently influenced diagnostic refinement or treatment modification. The absence of independent predictors in multivariate analysis further reflects the multifactorial and clinician-integrated nature of diagnostic decision-making in complex rheumatologic practice, rather than indicating methodological inadequacy.

The limitations of this study include that PET/CT indications were determined by clinical judgment rather than standardized criteria, potentially introducing selection bias; the cohort consisted primarily of diagnostically complex cases, limiting generalizability; and the retrospective design precluded control over treatment status, which may have influenced FDG uptake patterns. Additionally, not all diagnoses were histologically confirmed, and clinical follow-up served as a reference in some cases, introducing potential verification bias. Although a retrospective subset analysis demonstrated acceptable inter-observer agreement, image interpretation was primarily performed in a real-world, single-reader setting rather than in a prospective blinded multi-reader design. This may limit external reproducibility and generalizability. Quantitative analyses relied on SUVmax rather than more standardized vascular or tissue metrics. The lack of granular data regarding the exact type and timing of treatment modifications represents an important limitation of this study. Finally, heterogeneity in complementary imaging (MRI, CT angiography, Doppler ultrasound) limited direct comparison across modalities. Given the heterogeneity of our cohort, disease-specific conclusions should be interpreted cautiously. Certain subgroups, such as polymyalgia rheumatica and retroperitoneal fibrosis, included relatively small patient numbers, limiting statistical power and generalizability. Our study was not designed to provide disease-specific sensitivity or specificity estimates but rather to reflect real-world utilization patterns of PET/CT in a tertiary rheumatology setting. Therefore, the strongest inferences can be drawn for vasculitis, IgG4-related disease, and sarcoidosis, where diagnostic yield and clinical impact were more consistent. A further limitation relates to the inherent heterogeneity of the study cohort, which encompasses a broad spectrum of rheumatologic diagnoses reflecting real-world clinical practice. While this diversity strengthens the external validity of our findings, it also constrains subgroup-level inference. Several disease categories, including polymyalgia rheumatica (*n* = 8), crystal arthropathy (*n* = 4), and autoinflammatory disease (*n* = 4), are represented by small patient numbers that are insufficient to support definitive conclusions. Findings pertaining to these subgroups should therefore be interpreted as descriptive and hypothesis-generating, and prospective studies with larger disease-specific cohorts are needed to validate subgroup-specific diagnostic yields.

Our findings suggest that 18F-FDG PET/CT may serve as a valuable supportive tool in ruling out occult malignancy in patients with complex connective tissue diseases, particularly when combined with clinical and laboratory follow-up. However, the absence of FDG uptake does not entirely preclude the need for vigilance, and histopathological confirmation remains the gold standard for suspicious lesions. Despite these constraints, the study provides robust real-world evidence supporting PET/CT as a clinically impactful tool in rheumatologic evaluation.

## 5. Conclusions

18F-FDG PET/CT serves as a valuable adjunctive tool in the diagnostic evaluation and management of patients with systemic inflammatory diseases and complex rheumatologic presentations. It demonstrated the highest yield in vasculitis, polymyalgia rheumatica, IgG4-related disease, and sarcoidosis, while also serving as a valuable adjunctive tool in the assessment of possible malignancy, particularly in connective tissue diseases, when integrated with clinical, laboratory, and follow-up data. Integration of PET/CT with clinical and laboratory data enhances diagnostic precision and guides personalized treatment strategies. These findings support the role of PET/CT as an adjunctive imaging modality in real-world rheumatology practice, aiding diagnostic clarification and therapeutic decision-making in selected, clinically challenging scenarios.

## Figures and Tables

**Table 1:** Comparison of Pre-PET/CT Laboratory Parameters For Patients With or Without a Rheumatologic Diagnosis.

Laboratory Parameter	Without Rheumatologic Diagnosis	Rheumatologic Diagnosis	Total	*p*-Value
ACE (U/L)	48.4 (25.2–59.3)	31.5 (23.2–45.7)	37 (23.3–53.5)	0.705
ESR (mm/h)	51.5 (28–79)	40 (16–63)	42 (19–68)	**0.006**
CRP (mg/L)	31.6 (6–82.7)	13.8 (4.6–46.5)	16 (5–55.2)	**0.024**
Ferritin (ng/mL)	168 (32–341.5)	55.1 (21.7–153.9)	74 (24.6–194)	0.779
LDH (U/L)	189 (153–247)	205.5 (166–272)	196 (161–262)	0.120
AST (U/L)	19 (15–25)	20 (16–26)	20 (16–25)	0.442
ALT (U/L)	18 (13–28)	17 (13–27)	18 (13–27)	0.940
Creatinine (mg/dL)	0.7 (0.6–0.9)	0.6 (0.5–0.8)	0.7 (0.5–0.8)	0.397

ACE: Angiotensin-Converting Enzyme, ESR: Erythrocyte Sedimentation Rate, CRP: C-Reactive Protein, LDH: Lactate Dehydrogenase, AST: Aspartate Aminotransferase, ALT: Alanine Aminotransferase. Bold values indicate a statistically significant results (*p* < 0.05).

**Table 2:** Comparison of Laboratory Findings and Rheumatologic Subtypes Between Diagnosed and Undiagnosed Patients on PET/CT.

Parameter	Undiagnosed	Diagnosed	Total	*p*-Value
**Demographics**				
**Age (years), mean ± SD**	57.4 ± 14.6	58.7 ± 15.2	57.8 ± 14.7	0.844
**Sex, n** (%)				**0.015**
**Female**	135 (68.5)	44 (53.0)	179 (63.9)	
**Male**	62 (31.5)	39 (46.7)	101 (36.0)	
**Laboratory Parameters, median (IQR)**				
**ESR** (mm/h)	38 (16–61)	56 (32–79)	43 (19–69)	**<0.001**
**CRP** (mg/L)	14.3 (4.3–45)	33 (6.2–100)	16.9 (5–56.2)	**0.010**
**Ferritin** (ng/mL)	65 (25.7–178)	133 (23.7–316)	79.3 (25.1–195.9)	0.143
**LDH** (U/L)	196.5 (164–256)	200.5 (156.5–302)	197.5 (162–262)	0.120
**AST** (U/L)	20 (16–25)	19 (16–27)	20 (16–25.5)	0.073
**ALT** (U/L)	17 (13–26)	18 (13–29)	18 (13–27)	0.586
**Rheumatologic Disease Subtype, *n* (%)**				0.243
**Connective Tissue Disease**	59 (33.1)	11 (15.9)	70 (28.3)	–
**Vasculitis**	46 (25.8)	17 (24.6)	63 (25.5)	–
**Rheumatoid Arthritis**	24 (13.4)	4 (5.7)	28 (11.3)	–
**Spondyloarthritis**	17 (9.5)	4 (5.7)	21 (8.5)	–
**Other**	12 (6.7)	9 (13.0)	21 (8.5)	–
**Sarcoidosis**	5 (2.8)	9 (13.0)	14 (5.6)	–
**IgG4-Related Disease**	4 (2.2)	10 (14.4)	14 (5.6)	–
**Retroperitoneal Fibrosis**	4 (2.2)	4 (5.7)	8 (3.2)	–
**Polymyalgia Rheumatica**	7 (3.9)	1 (1.4)	8 (3.2)	–

ESR: Erythrocyte Sedimentation Rate, CRP: C-Reactive Protein, LDH: Lactate Dehydrogenase, AST: Aspartate Aminotransferase, ALT: Alanine Aminotransferase. Bold values indicate a statistically significant results (*p* < 0.05).

**Table 3:** Comparison of PET/CT Findings, Rheumatologic Subtypes, and Diagnostic Outcomes Based on CRP Levels (<5 mg/L vs. >5 mg/L).

Variables	CRP < 5 mg/L *n* (%)	CRP > 5 mg/L *n* (%)	Total	*p*-Value
**Sex**				0.023
**Female**	55 (75.3)	124 (59.9)	179 (63.9)	–
**Male**	18 (24.6)	83 (40.0)	101 (36.0)	–
**Rheumatologic Disease Subtype**				0.421
**Connective Tissue Disease**	26 (39.3)	44 (24.3)	70 (28.3)	–
**Vasculitis**	14 (21.2)	49 (27.0)	63 (25.5)	–
**Rheumatoid Arthritis**	4 (6.0)	24 (13.2)	28 (11.3)	–
**Spondyloarthritis**	3 (4.5)	18 (9.9)	21 (8.5)	–
**IgG4-Related Disease**	6 (9.0)	8 (4.4)	14 (5.6)	–
**Sarcoidosis**	7 (10.6)	7 (3.8)	14 (5.6)	–
**Other**	4 (6.0)	9 (4.9)	13 (5.2)	–
**Retroperitoneal Fibrosis**	1 (1.5)	7 (3.8)	8 (3.2)	–
**Crystal Arthropathy**	0 (0)	4 (2.2)	4 (1.6)	–
**Autoinflammatory Disease**	0 (0)	4 (2.2)	4 (1.6)	–
**Polymyalgia Rheumatica**	1 (1.5)	7 (3.8)	8 (3.2)	–
**PET/CT Indication**				0.848
**Diagnostic evaluation**	24 (32.8)	67 (32.3)	91 (32.5)	–
**Treatment response**	7 (9.5)	17 (8.2)	24 (8.5)	–
**Malignancy exclusion**	42 (57.5)	123 (59.4)	165 (58.9)	–
**Diagnosis with PET/CT**				0.272
**Yes**	18 (24.6)	65 (31.4)	83 (29.6)	–
**No**	55 (75.3)	142 (68.5)	197 (70.3)	–
**Rheumatologic Diagnosis by PET/CT**				0.562
**Yes**	12 (16.4)	28 (13.5)	40 (14.2)	–
**No**	61 (83.5)	179 (86.4)	240 (85.7)	–
**Non-Rheumatologic Diagnosis by PET/CT**				0.059
**Yes**	6 (8.2)	37 (17.8)	43 (15.3)	–
**No**	67 (91.7)	170 (82.1)	237 (84.6)	–
**Detection of Malignancy by PET/CT**				0.458
**Yes**	4 (5.4)	18 (8.6)	22 (7.8)	–
**No**	69 (94.5)	189 (91.3)	258 (92.1)	–
**Presence of lymphadenopathy in PET/CT Request**				0.173
**Yes**	44 (60.2)	104 (50.2)	148 (52.8)	–
**No**	29 (39.7)	103 (49.7)	132 (47.1)	–

**Table 4:** Comparison of Patients Diagnosed with Malignancy and Rheumatologic Disease on PET/CT.

Variable	Malignancy Diagnosis *n* (%)/Median (IQR)	Rheumatologic Diagnosis *n* (%)/Median (IQR)	*p*-Value
**Sex**			>0.05
**Female**	12 (54.5)	22 (55.0)	–
**Male**	10 (45.4)	18 (45.0)	–
**Age (years)**	65 (58–71)	59 (44.5–71)	>0.05
**PET/CT Parameters**			
**Highest SUVmax**	8.0 (6.1–10.6)	4.6 (3.3–7.2)	**0.016**
**Laboratory** **Parameters**			
**ESR** (mm/h)	54 (37–72)	52 (26–85)	0.534
**CRP** (mg/L)	37.6 (10.9–78)	34.2 (4.9–120.2)	0.386
**Ferritin** (ng/mL)	131.5 (18.8–239)	133 (28.6–261)	0.398
**LDH** (U/L)	222.5 (177.5–306.5)	178 (148–247)	0.081
**AST** (U/L)	20.5 (18–28)	19 (16.5–24)	0.443
**ALT** (U/L)	19 (15–28)	18.5 (14–28.5)	0.578
**Creatinine** (mg/dL)	0.7 (0.5–0.9)	0.8 (0.6–1.0)	0.463
**White Blood Cell** (×10^9^/L)	8.3 (5.9–9.5)	8.9 (6.8–10.8)	0.136
**Hemoglobin** (g/dL)	11 (9.7–12.7)	11.7 (10.8–12.4)	0.335
**Platelets** (×10^9^/L)	290.5 (213–442)	312 (238–363)	0.659

ESR: Erythrocyte Sedimentation Rate, CRP: C-Reactive Protein, LDH: Lactate Dehydrogenase, AST: Aspartate Aminotransferase, ALT: Alanine Aminotransferase. Bold values indicate a statistically significant results (*p* < 0.05).

**Table 5:** Comparison of PET/CT with Other Imaging Modalities.

Diagnosis (*n*)	Concordance with MRI (MRI+/PET/CT+)	Concordance with CT (CT+/PET/CT+)	Concordance with Doppler (Doppler+/PET/CT+)
Behçet’s disease (*n* = 5)	No data	1/1	1/1
Takayasu arteritis (*n* = 25)	6/15	1/2	7/18
Temporal arteritis (*n* = 18)	5/8	2/2	6/15
Others * (*n* = 8)	1/3	5/5	4/6

* Includes: 2 patients with Cogan’s syndrome, 4 patients with IgG4-related disease, and 2 patients with retroperitoneal fibrosis. MRI: Magnetic Resonance Imaging, CT: Computed Tomography, PET/CT: Positron Emission Tomography/Computed Tomography, Doppler: Doppler Ultrasonography.

## Data Availability

The datasets used and analyzed during the current study are available from the corresponding author on reasonable request.

## Supplementary Materials

The following supporting information can be downloaded at: https://www.mdpi.com/article/10.3390/jcm15051872/s1, Table S1: Clinical characteristics, PET/CT findings, and follow-up PET/CT of patients with sarcoidosis; Figure S1: PET/CT of a 23-year-old male patient undergoing evaluation for fever of unknown origin, showing diffuse bone marrow involvement (Adult-Onset Still’s Disease); Figure S2: PET/CT of a 27-year-old patient with rheumatoid arthritis (RA) performed due to persistent elevation of acute phase reactants, demonstrating widespread RA-related joint involvement; Figure S3: PET/CT of a 59-year-old female patient performed to support the diagnosis after Doppler ultrasonography revealed findings consistent with vasculitis in the subclavian arteries (Takayasu arteritis); Figure S4: PET/CT of a 72-year-old female patient performed due to elevated acute phase reactants, showing shoulder and hip girdle involvement consistent with polymyalgia rheumatica (PMR); Figure S5: PET/CT of a 31-year-old female patient with a preliminary diagnosis of IgG4-related disease, demonstrating bilateral tonsillar involvement; Figure S6: PET/CT of a 51-year-old male patient performed due to generalized lymphadenopathy and fever of unknown origin, revealing widespread lymph node involvement. (Diagnosis of sarcoidosis was confirmed by histopathology).

## Abbreviations

18F-FDG	Fluorine-18 Fluorodeoxyglucose.
ACE	Angiotensin-Converting Enzyme.
ALT	Alanine Aminotransferase.
AST	Aspartate Aminotransferase.
CRP	C-Reactive Protein.
CT	Computed Tomography.
CTD	Connective Tissue Disease.
csDMARD	Conventional Synthetic Disease-Modifying Antirheumatic Drug.
ESR	Erythrocyte Sedimentation Rate.
FUO	Fever of Unknown Origin.
IgG4-RD	Immunoglobulin G4-Related Disease.
IQR	Interquartile Range.
IUO	Inflammation of Unknown Origin.
LDH	Lactate Dehydrogenase.
MRI	Magnetic Resonance Imaging.
*n*	Number.
PET/CT	Positron Emission Tomography/Computed Tomography.
PMR	Polymyalgia Rheumatica.
RA	Rheumatoid Arthritis.
ROI	Region of Interest.
SD	Standard Deviation.
SjS	Sjögren’s Syndrome.
SLE	Systemic Lupus Erythematosus.
SpA	Spondyloarthritis.
SUVmax	Maximum Standardized Uptake Value.
USG	Ultrasonography.
WBC	White Blood Cell Count.
