# Supplementary material for: Clinical Utility of 18F-FDG PET/CT in Rheumatology: Diagnostic and Therapeutic Insights from a Ten-Year Real-World Cohort
Source: J Clin Med. 2026 Feb 28;15(5):1872. doi: 10.3390/jcm15051872 (PMC12986195; doi:10.3390/jcm15051872)
Supplement: Supplementary file 1 [file jcm-15-01872-s001.zip › jcm-4165057-supplementary.pdf]

Supplementary Table S1 Clinical characteristics, PET/CT findings, and follow-up PET/CT of patients with sarcoidosis

Patient (Age/Sex)	PET/CT Indication	Highest SUVmax (Region)	PET/CT Diagnosis	Histopathology	Reference Imaging	Follow-up PET/CT
(51/F)	FUO + generalized LAP	6.8 (Mediastinal/Hilar LAP)	Sarcoidosis	Positive (compatible)	PET/CT	–
(53/F)	Activity assessment	4.8 (Mediastinal/Hilar LAP)	Sarcoidosis	Not performed	Thorax CT	–
(33/M)	Mediastinal/Hilar LAP	9.7 (Mediastinal/Hilar LAP)	Sarcoidosis	Positive (compatible)	Thorax CT	–
(67/F)	Hypercalcemia + activity assessment	Physiological	Non-diagnostic	Not performed	Thorax CT	–
(55/F)	Generalized LAP	15.4 (Mediastinal/Hilar LAP)	Sarcoidosis	Positive (compatible)	PET/CT	–
(56/F)	Mediastinal/Hilar LAP	6.4 (Mediastinal/Hilar LAP)	Sarcoidosis	Positive (compatible)	PET/CT	–
(30/F)	Mediastinal/Hilar LAP	15.4 (Mediastinal/Hilar LAP)	Sarcoidosis	Positive (compatible)	PET/CT	–
(61/F)	Mediastinal/Hilar LAP	8.5 (Mediastinal/Hilar LAP)	Sarcoidosis	Not performed	PET/CT	Regression
(55/M)	FUO + generalized LAP	19.7 (Mediastinal/Hilar LAP)	Sarcoidosis	Positive (compatible)	PET/CT	–
(60/M)	Generalized LAP	7.9 (Mediastinal/Hilar LAP)	Sarcoidosis	Positive (compatible)	Thorax CT	Regression
(58/F)	Generalized LAP	9.7 (Rectosigmoid region)	Non-diagnostic	Positive (compatible)	Thorax CT	–
(66/F)	Mediastinal/Hilar LAP	5.7 (Mediastinal/Hilar LAP)	Sarcoidosis	Not performed	PET/CT	–
(75/F)	Mediastinal/Hilar LAP	4.3 (Mediastinal/Hilar LAP)	Sarcoidosis	Not performed	PET/CT	–
(47/F)	Generalized LAP	15.3 (Inguinal LAP)	Sarcoidosis	Positive (compatible)	PET/CT	Regression
(43/F)	Activity assessment	Physiological	Non-diagnostic	Positive (compatible)	Thorax CT	–
(38/F)	Mediastinal/Hilar LAP	5.4 (Mediastinal/Hilar LAP)	Sarcoidosis	Positive (compatible)	PET/CT	–

FUO: Fever of Unknown Origin, LAP: Lymphadenopathy, PET/CT: Positron Emission Tomography/Computed Tomography, SUVmax: Maximum Standardized Uptake Value, Thorax CT: Thoracic Computed Tomography

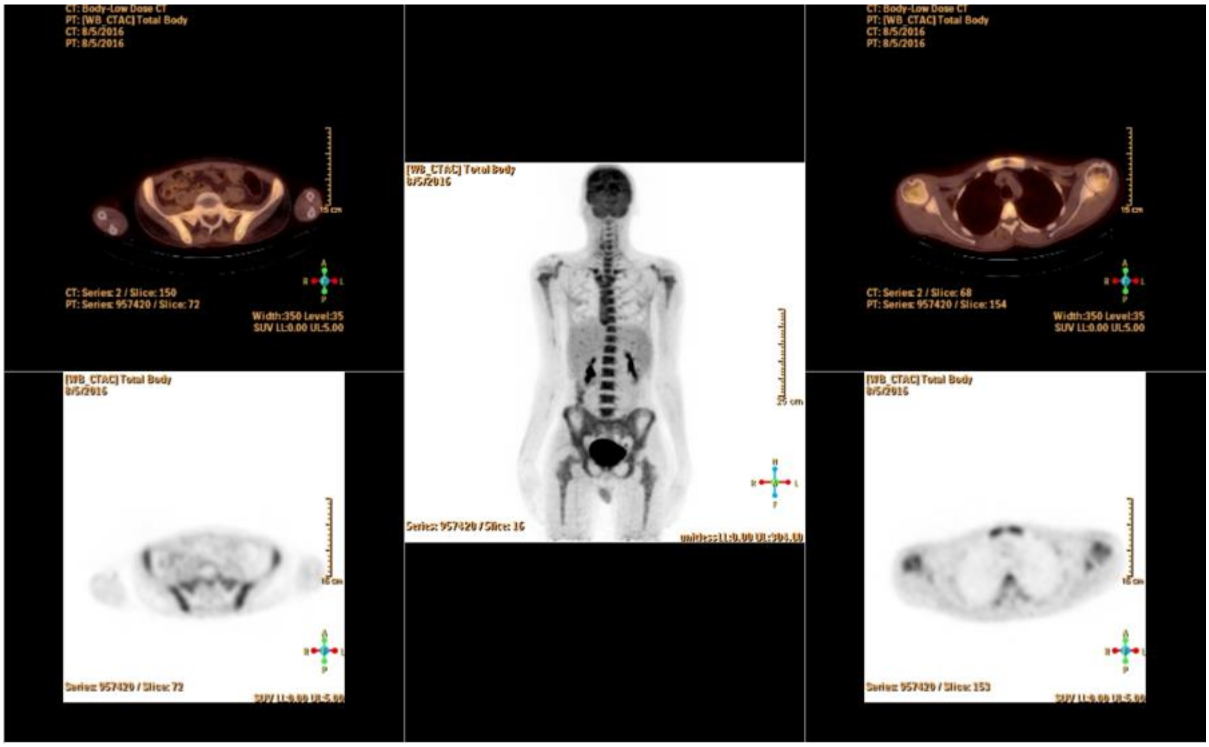

Supplementary Fig. S1 PET/CT of a 23-year-old male patient undergoing evaluation for fever of unknown origin, showing diffuse bone marrow involvement (Adult-Onset Still's Disease)

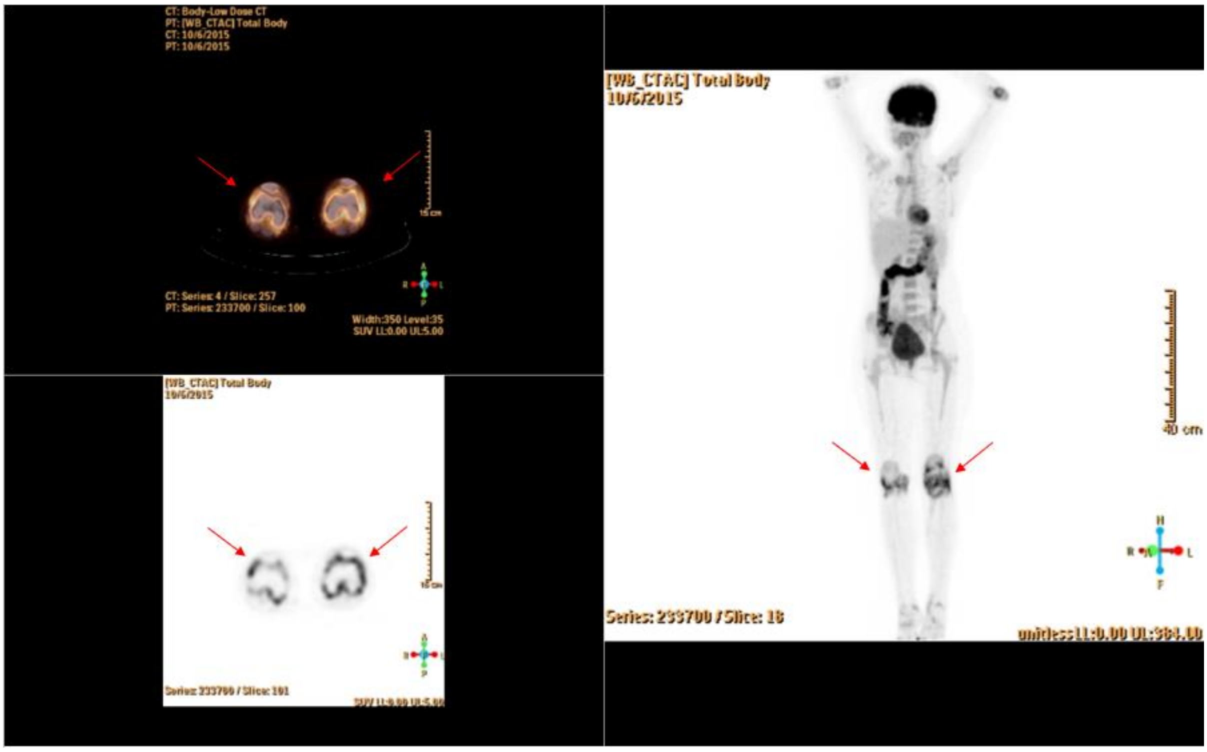

Supplementary Fig. S2 PET/CT of a 27-year-old patient with rheumatoid arthritis (RA) performed due to persistent elevation of acute phase reactants, demonstrating widespread RA-related joint involvement

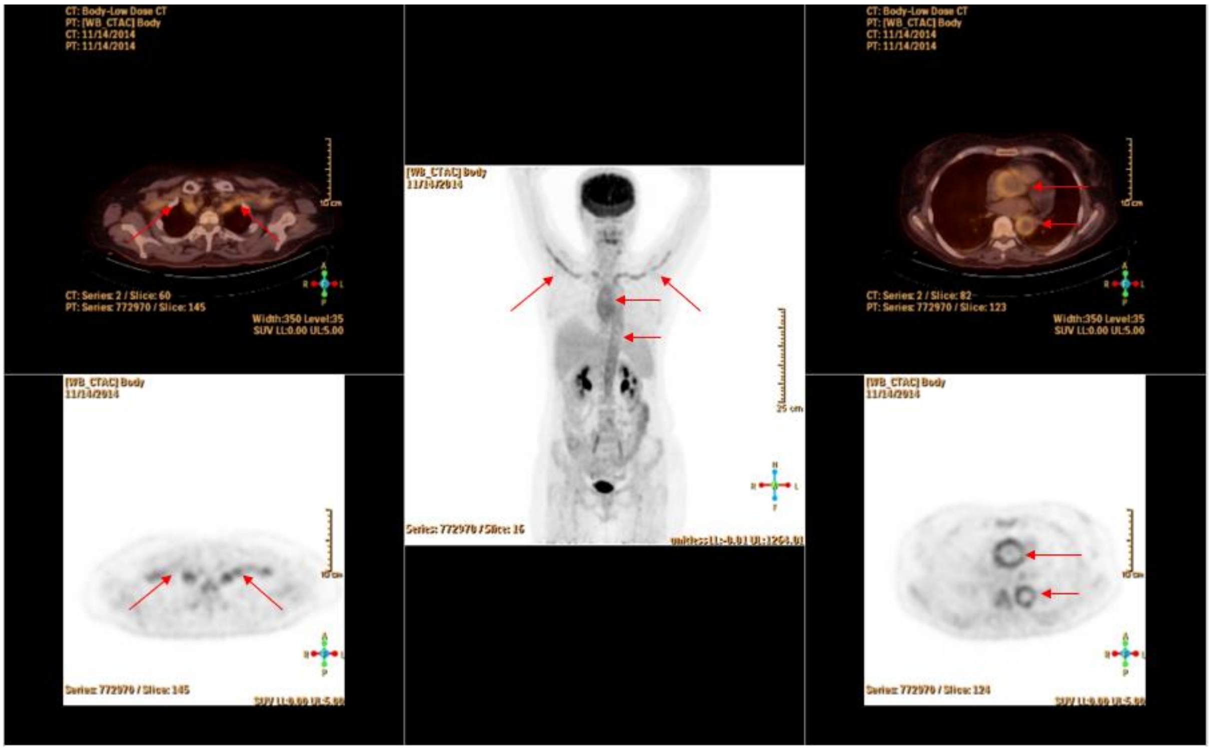

Supplementary Fig. S3 PET/CT of a 59-year-old female patient performed to support the diagnosis after Doppler ultrasonography revealed findings consistent with vasculitis in the subclavian arteries (Takayasu arteritis)

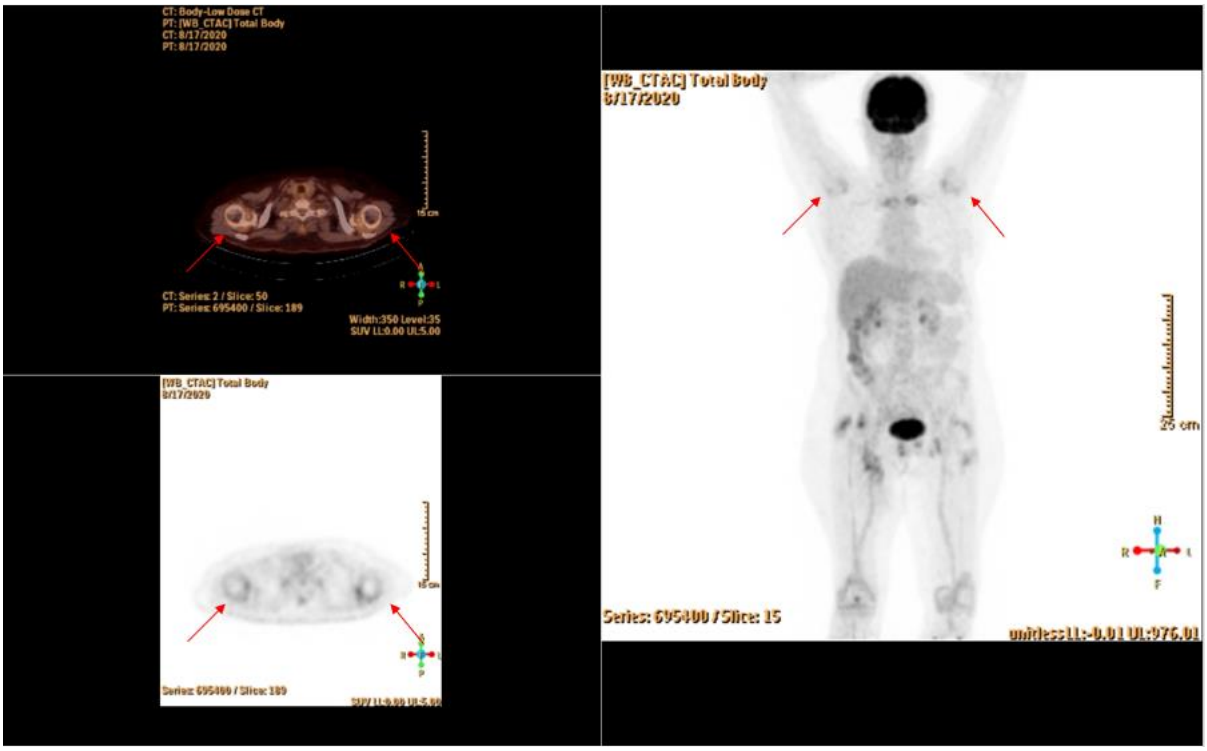

Supplementary Fig. S4 PET/CT of a 72-year-old female patient performed due to elevated acute phase reactants, showing shoulder and hip girdle involvement consistent with polymyalgia rheumatica (PMR)

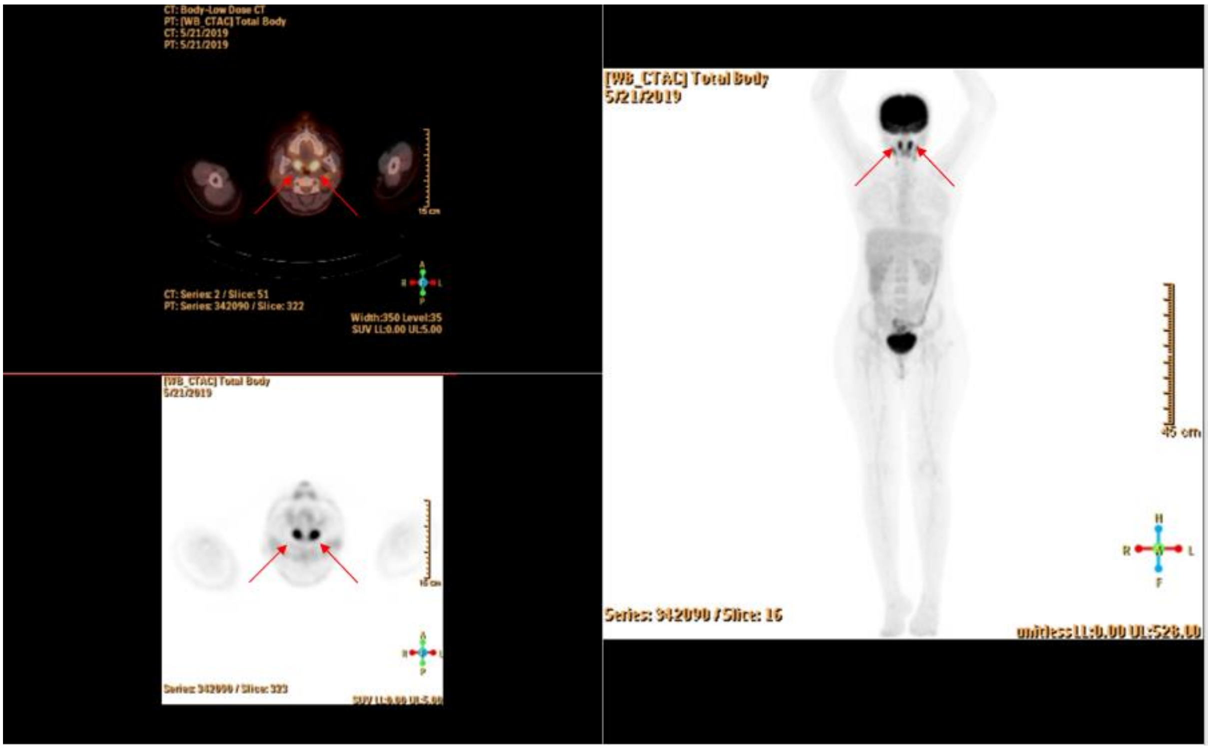

Supplementary Fig. S5 PET/CT of a 31-year-old female patient with a preliminary diagnosis of IgG4-related disease, demonstrating bilateral tonsillar involvement

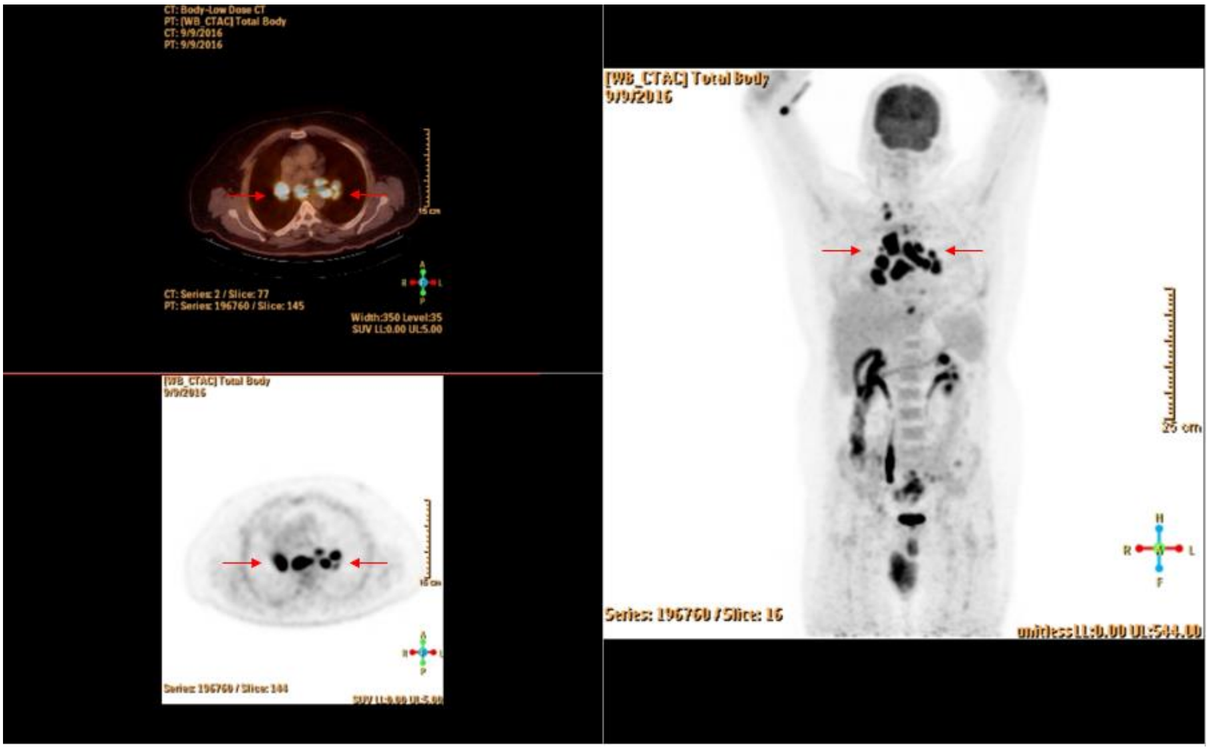

Supplementary Fig. S6 PET/CT of a 51-year-old male patient performed due to generalized lymphadenopathy and fever of unknown origin, revealing widespread lymph node involvement. (Diagnosis of sarcoidosis was confirmed by histopathology)
